# Usefulness of Point-of-Care Testing for Respiratory Viruses in a Pediatric Emergency Department Setting

**DOI:** 10.3390/jcm13237368

**Published:** 2024-12-03

**Authors:** Tommaso Bellini, Elena Fueri, Clelia Formigoni, Marcello Mariani, Giovanna Villa, Martina Finetti, Marta Marin, Elena De Chiara, Anna Bratta, Barbara Vanorio, Federica Casabona, Carlotta Pepino, Elio Castagnola, Emanuela Piccotti, Andrea Moscatelli

**Affiliations:** 1 Paediatric Emergency Room and Emergency Medicine Unit, Emergency Department, IRCCS Istituto Giannina Gaslini, via G. Gaslini, 5, 16147 Genoa, Italy; elenafueri@gaslini.org (E.F.); giovannavilla@gaslini.org (G.V.); martinafinetti@gaslini.org (M.F.); martamarin@gaslini.org (M.M.); carlottapepino@gaslini.org (C.P.); emanuelapiccotti@gaslini.org (E.P.); 2 Department of Neuroscience, Rehabilitation, Ophthalmology, Genetics, Maternal and Child Health (DINOGMI), University of Genoa, 16147 Genoa, Italy; 3805091@studenti.unige.it (C.F.); 5302947@studenti.unige.it (E.D.C.); 5726141@studenti.unige.it (A.B.); 5749021@studenti.unige.it (B.V.); 3907391@studenti.unige.it (F.C.); 3 Infectious Diseases Unit and COVID Hospital, Department of Pediatrics, IRCCS Istituto Giannina Gaslini, 16147 Genoa, Italyeliocastagnola@gaslini.org (E.C.); 4 Pediatric and Neonatal Intensive Care Unit, Emergency Department, IRCCS Istituto Giannina Gaslini, 16147 Genoa, Italy; andreamoscatelli@gaslini.org

**Keywords:** adenovirus, fever, influenza, overcrowding, pediatric emergency department, point of care, respiratory syncytial virus

## Abstract

**Background**: Respiratory tract infections (RTIs) are a leading cause of pediatric emergency department (PED) visits, especially in children under five. These infections are primarily viral, complicating diagnosis and management. This study assesses the impact of point-of-care (POC) rapid diagnostic tests for respiratory viruses on clinical and economic outcomes in a PED setting. **Materials and Methods:** A retrospective analysis of 1396 POC tests for RSV, adenovirus, and influenza A/B was conducted in the PED of the Giannina Gaslini Institute, Genoa, Italy, from December 2022 to April 2024. Demographics, blood tests, admissions, and readmission rates were evaluated. Statistical analyses were performed using appropriate tests for categorical and continuous variables. **Results:** Of the tests, 31.5% were positive for at least one virus. Positive patients were younger and had higher hospitalization rates (50.7% vs. 39.9%) but fewer blood tests (38.2% vs. 51.7%). Economic analysis indicated lower costs for virus-positive patients. RSV-positive patients showed a significant association with higher hospitalization rates (67.8%) and readmission within 72 h. **Conclusions:** POC testing significantly improves patient management in PEDs by enabling rapid diagnoses, reducing unnecessary tests and hospitalizations, and guiding appropriate treatment. This approach supports better resource allocation, crucial during peak seasons, and has implications for reducing antibiotic use and resistance. Further research is warranted to explore long-term impacts on patient outcomes and healthcare efficiency.

## 1. Introduction

Respiratory tract infections (RTIs) represent one of the main reasons for visits to pediatric emergency departments (PEDs) [1,2,3,4]. These infections manifest frequently during the initial decade of life, accounting for up to 50% of PED admissions in children under 5 years of age [1,4,5,6,7,8,9].

RTIs are predominantly of viral etiology, with rhinovirus, influenza virus, parainfluenza virus, adenovirus, and respiratory syncytial virus (RSV) being the most prevalent causative agents [1,2,8,10,11,12]. However, the nonspecific clinical presentation may complicate the differential diagnosis with bacterial infections, frequently necessitating a blood test, brief intensive observation, or hospital admission [4,5,6,8,12,13,14]. Furthermore, in recent years, novel laboratory techniques have facilitated the discovery of new respiratory viruses, such as human metapneumovirus and human bocavirus, which may account for up to 10% of total viral infections [1,10,11,15]. Consequently, the differential diagnosis between viral and bacterial etiologies is crucial [4,16].

While reverse transcription–quantitative polymerase chain reaction (RT-qPCR) remains the gold standard for detecting respiratory infectious diseases, it may present with several drawbacks. The technique is not only costly but also requires considerable time to produce results. Additionally, it necessitates the expertise of trained personnel, making it challenging to process high volumes of samples outside of laboratory settings [4]. The implementation of point-of-care (POC) rapid diagnostic tests has the potential to enhance early etiological diagnosis, thereby optimizing clinical management, reducing diagnostic time, and promoting more efficient utilization of healthcare resources, especially during peak periods when emergency department visits significantly increase and hospital bed availability is limited [1,3,4,8,11,15]. Moreover, early identification of a viral etiology through POC tests would facilitate the limitation of inappropriate antibiotic use, thereby avoiding unnecessary treatments and contributing to the reduction in antibiotic resistance, in accordance with antibiotic-sparing strategies [1,7,8,9,16].

The present study aimed to assess the impact of POC diagnostic tests on the management of pediatric patients with respiratory conditions in PED. The analysis focused on the benefits from both a clinical perspective, including the optimization of human resources, reduction in diagnostic times, utilization of blood tests, and hospitalization rates, and from an economic perspective, examining the efficiency of healthcare resource allocation and the sustainability of associated costs.

## 2. Materials and Methods

### 2.1. Study Design and Setting

A retrospective analysis was conducted on POC antigen tests for RSV, adenovirus, and influenza A/B virus via nasopharyngeal swabs, performed in the PED of the Giannina Gaslini Institute in Genoa, Italy, during the periods from 12 May 2022 to 4 February 2023 and 12 June 2023 to 4 February 2024. All swabs were performed with Standard F antigen POCs produced by Relab, SD BiosensorTM. Table 1 summarizes the specificity and sensitivity characteristics of the POCs used [17]. The tests have a rapid turnaround time of 10 min and were performed on demand at bedside, with no transport time to a laboratory for testing. The point-of-care test is highly user-friendly, requiring no specific expertise, and can be performed by any healthcare personnel after a brief demonstration.

### 2.2. Study Population

All POC tests conducted on febrile children aged 0–18 years with a temperature exceeding 38 °C and presenting symptoms attributable to airway infections were included in the study. All patients who underwent POC testing were confirmed negative for SARS-CoV-2 upon admission to the PED. The present investigation did not employ any additional exclusion criteria.

### 2.3. Data Collection

For each patient tested, sex, age, blood test execution, and management in terms of admission or discharge were evaluated, as well as the rate of return to the PED within 72 h of discharge. The cost of a single test was less than 10 euros, and no additional personnel expenses were incurred as healthcare workers in the PED performed the test. Moreover, the economic implications of patient management were analyzed by estimating the costs associated with hospitalization and blood tests. The average cost of hospitalization was calculated based on data related to suspected diagnoses categorized under the Diagnosis Related Groups (DRGs) defined by the Italian Health System. For blood tests, the cost was determined using ministerial tables that provide standardized pricing for individual tests.

### 2.4. Statistical Analysis

Categorical variables are expressed as absolute frequencies and percentage. Continuous variables are presented as the mean and standard deviation or the median and interquartile range when not normally distributed. Kruskal–Wallis test or Mann–Whitney U-test for continuous variables, and chi-square or Fisher’s exact test for categorical variables, were used to access differences between the groups. Statistical significance was set at *p* < 0.05, and all values were determined using two-tailed tests. All statistical analyses were conducted using GraphPad Prism version 9.1.0 for Windows (GraphPad Software, San Diego, CA, USA, www.graphpad.com, accessed on 1 October 2024) or IBM SPSS Statistics for Windows Version 21.0 (IBM Corp., Armonk, NY, USA).

## 3. Results

### 3.1. Total Tests

A total of 1396 tests from 1178 patients were analyzed, of which 31.5% (440/1396) yielded positive results for one of the viruses examined (RSV, adenovirus, or influenza A/B).

### 3.2. Adenovirus Tests

A total of 291 adenovirus tests were performed, with a positivity rate of 10.6%. No demographic differences were observed between the positive and negative groups. Notably, positive patients underwent blood tests significantly less frequently than negative patients (29.0% vs. 67.3%, *p* < 0.0001) and exhibited significantly lower hospitalization rates (12.9% vs. 41.9%, *p* = 0.0015), resulting in substantially lower mean costs (274.79 vs. 1085.88 euros, *p* = 0.0007). There were no significant differences in the readmission rates between the two groups following home discharge during the initial PED evaluation. The main findings are presented in Table 2.

### 3.3. Influenza A/B Tests

Among 458 tests for influenza A/B, a positivity rate of 24.8% was observed. No demographic disparities were evident between the positive and negative groups. Consistent with the findings for adenovirus, positive patients underwent blood tests less frequently (32.4% vs. 54.3%, *p* < 0.0001) and exhibited lower hospitalization rates (16.6% vs. 31.4%, *p* = 0.0023), resulting in significantly reduced mean costs (351.85 vs. 815.61 euros, *p* = 0.0001). No significant differences in readmission rates within 72 h were observed between the two groups. The main results are presented in Table 3.

### 3.4. RSV Tests

A total of 647 tests for RSV were performed, of which 45.6% were positive. No gender differences were observed. However, the RSV-positive patients were significantly older than their negative counterparts (9.2 vs. 7.8 months, *p* = 0.037). RSV-positive patients exhibited significantly higher hospitalization rates (67.8% vs. 46.8%, *p* < 0.0001) than RSV-negative patients, although there was no difference in the rate of blood tests performed. The mean cost of care was significantly higher for positive patients (*p* = 0.0053). Lastly, RSV-positive patients discharged after the initial PED assessment demonstrated a higher rate of readmission within the subsequent 72 h (*p* < 0.0001). The main results are presented in Table 4.

## 4. Discussion

Physicians are often unable to accurately predict which child will have a positive test for one of the most common viruses based on history and physical findings alone [2]. Consequently, etiological diagnosis based on clinical presentation alone is challenging and may result in management delays [4,13]. Furthermore, the SARS-CoV-2 pandemic, and previously the H1N1 influenza pandemic, have demonstrated that the necessity of performing massive screening in the population may overwhelm the capacity of microbiology laboratories. Hence, rapid tests that can be performed within minutes must be available [10,18,19,20]. Indeed, physicians require the appropriate test, for the appropriate patient, performed at the appropriate time [2,10].

The findings of the present study underscore the significant role that POC testing for adenovirus, influenza A/B, and RSV may play in managing patients in the PED. These viruses may represent up to 40% of total viral infections, and the ability to rapidly diagnose them at the bedside could offer several advantages, including reducing the need for invasive testing, informing the decision-making process regarding hospitalization, and ultimately reducing healthcare costs [1,2,6,13,15,21,22,23,24]. Our data, in fact, confirm that the integration of these POC antigen tests, costing less than 10 euros each, could provide an efficient 24/7 solution to avoid unnecessary hospital admissions and diagnostic procedures, such as blood tests or radiation exposure [5,6,13,21,23,25]. This is particularly relevant in pediatric patients, where blood draws can be distressing for the patient and their family. A reduction in such procedures improves the overall patient experience and allows healthcare professionals to allocate time to more critical tasks, thus enhancing the overall efficiency of PEDs. Moreover, given that the POC tests are performed on demand, the efficiency in the PED may be optimized by eliminating the transport time to the laboratory for testing that is still deemed for traditional multiplex RT-qPCR tests [4,10,11,15,18,23,24,26,27]. This may be also applicable in low- and middle-income countries, where child mortality due to common respiratory infections is higher than in high-income countries [10,21,22,24]. However, in our cohort we were not able to provide data on the socioeconomic and income status of the enrolled patients, as it cannot be directly extrapolated from our medical records.

The ability to promptly distinguish between bacterial and viral infections enables clinicians to avoid unnecessary antibiotic prescriptions, a consideration of particular importance worldwide given the ongoing global concern surrounding antimicrobial resistance [1,2,5,7,8,9,13,14,16,27,28]. This is especially relevant in adenovirus infections, which can mimic bacterial infections and are characterized by high-grade, prolonged fever and elevated levels of acute-phase inflammatory markers [15,22]. Moreover, a positive POC test could also influence the decision to discontinue antibiotic therapy in patients with persistent fever that is unresponsive to antibiotics [28,29]. In the present case series, no patient who tested positive for adenovirus or influenza A/B was discharged with antibiotic therapy. Moreover, in children at higher risk of complications, such as those younger than 2 years of age, immunosuppressed, or with chronic medical conditions, a positive POC test for influenza A/B may lead to a rapid initiation of antiviral therapy, given that the therapy should be initiated within 48 h of illness onset [2,3,9,13,16,19,24,26,27,28]. This is especially relevant during winter months, a period characterized by high patient volumes in the PED, where efficient resource allocation is critical [10,25]. Thus, the ability to safely discharge patients with viral infections, without increasing the risk of readmission, optimizes the utilization of healthcare resources [10,11,13,15]. Furthermore, it should be noted that the result speed, of approximately 15 min, can also expedite the boarding time in the PED for each patient, reducing the risk of overcrowding during peak epidemic months [6,8,10,15,24,26,30].

The results for RSV-positive patients were consistent with the expectations delineated in the current literature [22]. RSV is well established for its association with severe bronchiolitis and respiratory distress in young children, frequently necessitating more intensive clinical management, including hospitalization. Rapid RSV testing in the emergency setting is crucial for triaging patients who may require higher levels of care [7,22]. RSV etiology in bronchiolitis is associated with a more severe clinical course compared to other causative agents of bronchiolitis [31]. Therefore, rapid RSV testing in PED may facilitate the identification of patients at elevated risk of clinical deterioration who may require more intensive clinical management [7]. Consequently, a positive RSV test result may increase the probability of hospitalization [31]. Furthermore, this study corroborates that a positive RSV result may reduce the necessity for additional diagnostic investigations, such as chest X-rays or blood cultures, and minimizes the utilization of unnecessary antibiotics [7,9,12,16,23,24,27]. This is particularly pertinent, as empirical antibiotic use remains a prevalent practice in pediatric bronchiolitis, despite clear viral etiologies [4,5,6]. Additionally, RSV positivity could lead to cohort isolation of patients in the event of hospitalization, preventing exposure to the virus in RSV-negative or unknown patients [6,22]. However, not all studies agree that the use of POC tests can actually reduce antibiotic prescription and future studies are still needed to clarify results that appear conflicting [7,9,16,23,24,27].

Interestingly, RSV-positive patients were older than those who tested negative for the same virus and this result may have various interpretations. Our study started in 2022, when the lockdown measures for the SARS-CoV-2 pandemic were progressively reduced, so it is reasonable to think that older children were infected by RSV due to a reduced recirculation of the virus in the previous epidemic season [31,32]. In addition, it has been widely described how the highest incidence of clinically relevant infection is more frequent between 3 and 6 months of life, compared to very young infants, probably due to a progressive decrease in maternal antibody protection together with an immaturity of the immune system [33,34,35].

Finally, it should be remembered that both physicians and parents may derive satisfaction from the ability to ascribe a specific diagnosis to a child’s illness, thereby avoiding the frustration associated with the statement “it is probably just a virus” and showing a greater confidence in diagnosis and in isolation strategies [1,27].

### Limits

This study did not consider the risk of coinfection with multiple viruses concurrently, given the exclusion of SARS-CoV-2 positive patients upon PED admission. However, all patients were deemed negative based on a negative test for the three viruses examined; alternatively, they could have been positive for another untested virus, such as human metapneumovirus, human bocavirus, or parainfluenza virus. Moreover, concurrent bacterial infections, which, although infrequent, can coexist with viral infections, were not considered. Lastly, while the POC antigen test demonstrates high specificity across multiple pathogens, its sensitivity varies depending on the target virus and it is comparable with other tests [4,12,24]. For instance, while sensitivity for influenza reaches an impressive 97.0%, it is slightly lower for adenovirus (83.3%) and RSV (90.5%). This variability highlights the need for cautious interpretation of negative results, particularly in clinical scenarios with a high pretest probability, and suggests that confirmatory testing may be beneficial in certain cases. Moreover, studies with larger sample sizes may be needed to confirm the data obtained. Finally, as stated before, we were not able to collect data on the socioeconomic and income status of the enrolled patients; this could represent a starting point for a future prospective study to evaluate the role of family social status in impacting the epidemiology of the viruses examined.

## 5. Conclusions

POC tests for adenovirus and influenza A/B in an emergency department setting facilitate early diagnosis and safe discharge. Furthermore, a positive RSV POC test may indicate to the physician those patients who may be at a higher risk and consequently require hospital admission. Overall, the implementation of rapid antigen testing for respiratory viruses in PEDs not only enhances patient care by enabling timely and accurate diagnoses, but also facilitates the optimization of healthcare resources, reducing the rate of blood tests performed, the hospitalization rate, and the prescription of unnecessary antibiotics. Future studies should further investigate the long-term impacts of POC testing on patient outcomes, particularly in terms of antibiotic resistance patterns and healthcare cost-effectiveness during peak respiratory virus seasons. Moreover, these POC tests may also be applicable in ambulatory settings. This evidence supports the integration of these diagnostics as cornerstones in the management of pediatric respiratory infections, promoting both clinical efficacy and economic sustainability.

## Figures and Tables

**Table 1:** Sensitivity and specificity characteristics of the POC tests.

	Sensitivity	Specificity
Influenza	97.0%	97.6%
Adenovirus	83.3%	95.5%
RSV	90.5%	92.2%

**Table 2:** Main results comparing adenovirus positive and negative tests.

	Adenovirus Tests *n* = 291	Positive Tests *n* = 31	Negative Tests *n* = 260	*p*
Sex, male (%)	163 (56)	17 (54.8)	146 (56.1)	0.88
Age in months, median (IQR)	77.8 (35.4–178.8)	70.8 (36.0–113.3)	82.3 (35.5–200.3)	0.14
Blood Tests, yes (%)	184 (63.2)	9 (29.0)	175 (67.3)	<0.0001
Admission, yes (%)	113 (38.8)	4 (12.9)	109 (41.9)	0.0015
Readmission, yes (%) ^1^	12 (6.7)	1 (3.7)	11 (7.3)	0.69
Cost in euros, Median (IQ 25–75)	61.15 (0.00–2553.15)	61.15 (0.00–2553.15)	0.00 (0.00–61.15)	0.0007

^1^ The percentage of ED readmissions was calculated among patients discharged home after their initial ED evaluation.

**Table 3:** Main results comparing Influenza A/B positive and negative tests.

	Influenza A/B Tests *n* = 458	Positive Tests *n* = 114	Negative Tests *n* = 344	*p*
Sex, male (%)	257 (56.1)	59 (51.7)	198 (57.5)	0.27
Age in months, median (IQR)	93.9 (42.3–205.1)	100.8 (54.6–175.1)	90.3 (38.2–217.2)	0.57
Blood Tests, yes (%)	224 (48.9)	37 (32.4)	187 (54.3)	<0.0001
Admission, yes (%)	127 (27.7)	19 (16.6)	108 (31.4)	0.0023
Readmission, yes (%) ^1^	19 (5.7)	8 (8.4)	11 (4.7)	0.19
Cost in euros, Median (IQ 25–75)	61.15 (0.00–2053.15)	61.15 (0.00–2553.15)	0.00 (0.00–61.15)	0.0001

^1^ The percentage of ED readmissions was calculated among patients discharged home after their initial ED evaluation.

**Table 4:** Main results comparing RSV positive and negative tests.

	RSV Tests *n* = 647	Positive Tests *n* = 295	Negative Tests *n* = 352	*p*
Sex, male (%)	376 (58.1)	169 (57.3)	207 (58.8)	0.69
Age in months, median (IQR)	8.4 (4.5–15.1)	9.2 (5.0–14.8)	7.8 (4.1–15.3)	0.037
Blood Tests, yes (%)	254 (39.2)	122 (41.3)	132 (37.5)	0.33
Admission, yes (%)	365 (56.4)	200 (67.8)	165 (46.8)	<0.0001
Readmission, yes (%) ^1^	42 (14.9)	28 (29.4)	14 (7.5)	<0.0001
Cost in euros, Median (IQ 25–75)	1845.00 (0.00–2288.00)	61.15 (0.00–2349.15)	0.00 (0.00–1906.15)	0.0053

^1^ The percentage of ED readmissions was calculated among patients discharged home after their initial ED evaluation.

## Data Availability

The datasets used and analyzed in this paper are available from the corresponding author on reasonable request.
